# Immigrant and minority parents' experiences in a neonatal intensive care unit: A meta‐ethnography review

**DOI:** 10.1111/jocn.17402

**Published:** 2024-08-23

**Authors:** Suza Trajkovski, Mahmoud A. Al‐Dabbas, Shanti Raman, Nicolette Giannoutsos, Margaret Langman, Virginia Schmied

**Affiliations:** ^1^ School of Nursing and Midwifery Western Sydney University Penrith New South Wales Australia; ^2^ NICM Health Research Institute, Western Sydney University Penrith New South Wales Australia; ^3^ South Western Sydney Local Health District Liverpool New South Wales Australia; ^4^ UNSW Kensington New South Wales Australia; ^5^ CNC, Neonatal Intensive Care Unit, Liverpool Hospital, South Western Sydney Local Health District Liverpool New South Wales Australia; ^6^ Neonatal Intensive Care Unit, Liverpool Hospital, South Western Sydney Local Health District Liverpool New South Wales Australia

**Keywords:** cross‐cultural care, meta‐ethnography, migrant, minority families, neonatal unit, refugee

## Abstract

**Aims:**

To examine immigrant and minority parents' experiences of having a newborn infant in the neonatal intensive care unit and explore healthcare professionals' experiences in delivering care to immigrant and minority families.

**Design:**

A meta‐ethnographic review informed by eMERGe guidelines.

**Methods:**

We conducted a systematic literature review. Studies were included if they explored immigrant or minority parent experiences in neonatal intensive care units and health professional experiences delivering care to immigrant and minority families in neonatal intensive care. Reporting followed ENTREQ guidelines.

**Data Sources:**

Database searches included CINAHL, MEDLINE, PubMed, PsycINFO, Scopus and Google Scholar. Boolean search strategies were used to identify qualitative studies. No limitations on commencement date; the end date was 23rd August 2022. PRISMA guidelines used for screening and article quality assessed using Joanna Briggs Institute criteria for qualitative studies.

**Results:**

Initial search yielded 2468 articles, and nine articles met criteria for inclusion. Three overarching themes were identified: (1) Overwhelming Emotions, (subthemes: Overwhelming Inadequacy; Cultural Expressions of Guilt; Not Belonging), (2) Circles of Support, (subthemes: Individual Level‐Spirituality; External Level‐Connecting with Family; Structured Peer‐to‐Peer Support), (3) Negotiating Relationships with Healthcare Professionals (subthemes: Connecting; Disconnected; Linguistic Barriers). Interactions between healthcare professionals and immigrant and minority parents were the strongest recurring theme.

**Conclusions:**

There can be a mismatch between immigrant and minority families' needs and the service support provided, indicating improvements in neonatal intensive care are needed. Despite challenges, parents bring cultural and family strengths that support them through this time, and many neonatal intensive care staff provide culturally respectful care.

**Implications for the Profession and/or Patient Care:**

Professionals should be encouraged to identify and work with family strengths to ensure parents feel supported in the neonatal intensive care unit. Findings can inform policy and practice development to strengthen health professionals capabilities to support immigrant and minority families in neonatal units.

**Reporting Method:**

The Preferred Reporting Items for Systematic Reviews and Meta‐Analyses checklists were used to report the screening process.

**What does this paper contribute to the wider global community?**

- This review can provide a foundation for further research exploring immigrant and minority NICU care in both high and low‐resource countries.
- Findings from this review can also help guide and strengthen partnerships between healthcare professionals and families from immigrant and minority populations experiencing NICU care.
- Researchers, healthcare managers, and policymakers could build upon the findings to develop targeted interventions and policies for supporting immigrant and minority families in the NICU.

## 1 INTRODUCTION

The neonatal intensive care unit (NICU) provides specialised medical care and often life‐saving treatment to sick or premature infants. Preterm birth or the birth of a sick‐term infant often leads to separation from family during the infant's admission to a NICU. Parents with an infant requiring neonatal care report experiencing stress, anxiety, and depression due to and uncertainty of their infants' survival and risk of future disabilities and their inability to perform infant care activities (Roque et al., 2017; Shovers et al., 2021).

Increasingly, research has demonstrated the negative consequences of separating infants from parents soon after birth, including altered emotional and cognitive development of the infant, acute stress, and post‐traumatic stress disorders for parents (Pineda et al., 2018; Roque et al., 2017; Schecter et al., 2020). The intensive care provided by healthcare professionals for the infant as well as the unit policies and interactions between parents and healthcare professionals may also result in parents feeling they do not have ‘ownership’ of their infant (Vazquez & Cong, 2014). New guidelines strongly recommend that kangaroo mother care be initiated as soon as possible after birth including preterm or low‐birth‐weight infants as it significantly improves survival rates (World Health Organisation, 2022). Kangaroo care is skin‐to‐skin contact between the parent/carer and infant by placing the undressed infant on the parent/carer's bare chest. Increasing research demonstrates the positive outcomes of facilitating continuous parent‐infant contact in NICU (Arya et al., 2021; Zhu et al., 2023).

The experience of NICU admission and separation is potentially more difficult for immigrant and minority parents for whom the health system is foreign. In some instances, limited proficiency in the local language may hamper their ability to communicate with healthcare professionals, understand their infant's condition and their opportunity to be involved in their infant's care or treatment (Shovers et al., 2021). Family‐centred care (FCC) is a model of care that requires developing respectful and mutually beneficial partnerships between healthcare professionals, patients, and families in planning, delivering, and evaluating healthcare. The Institute for Patient and Family‐Centered Care (2018) emphasises that within the philosophy of FCC is the need to provide care honouring the racial, ethnic, and cultural diversity of families and to design health care that is culturally appropriate and responsive to infant and family needs.

Previous studies have demonstrated the challenges that immigrant and minority mothers experience accessing and engaging in services during pregnancy and birth, noting that these are influenced by the, at times, inflexible nature of the healthcare system as well as parent‐embedded cultural values, traditions and expectations (Fair et al., 2020; Higginbottom et al., 2014). Over the past decade, a small number of qualitative studies have explored the experiences of immigrant and minority parents with infants in a NICU. This offers the opportunity to synthesise these existing studies to capture the aspects of experience that are common to this parent population, to identify research gaps, and where possible, to apply to the findings to inform policy in nurseries. Meta‐ethnography was selected as the most appropriate approach to synthesise this literature.

Terms such as ‘migrants’, ‘asylum seekers’, ‘refugees’ or ‘foreign‐born’ are often used interchangeably in the literature. For this review, the broad term ‘immigrant’ will be used. An immigrant can be defined as a ‘person living in a country other than that of his or her birth’ (Migration Policy Institute, 2024, p. 1). Similarly, the consensus of what constitutes a minority group is lacking; therefore, for this review, a minority group is defined as ‘an ethnic, religious or linguistic minority is any group of persons that constitutes less than half of the population in the entire territory of a State whose members share common characteristics of culture, religion or language, or a combination of any of these’ (United Nations Human Rights Office, 2024, p. 1). The focus of this review is on immigrant and minority families who differ from the majority population of the country they are now living in because of first language, culture, race or religion.

### 1.1 Aim

In this review, we aimed to examine immigrant and minority parents' experiences of having a newborn infant in the neonatal intensive care unit and healthcare professionals' experiences in delivering care to immigrant and minority families in the NICU.

## 2 METHOD

### 2.1 Design

Noblit and Hare's (1988) meta‐ethnographic approach was used to guide this synthesis of qualitative literature. A meta‐ethnographic approach was used as it can increase understanding of a phenomenon, increase the relevance of findings from single qualitative studies to broader contexts, identify directions for future research and can provide evidence to inform health care policy and practice development (France et al., 2019). Meta‐ethnography allows the reviewer to re‐interpret conceptual data, such as concepts, themes, or metaphors, created by the authors of primary studies using a step‐by‐step process to extend findings of individual study accounts, which may be specific to the cultural needs of neonates and their families and health professionals providing care (France et al., 2019). Noblit and Hare (1988) outlined seven phases conducted in a series of overlapping, parallel and iterative processes to systematically compare studies. Using the approach of Noblit and Hare (1988) and eMERGEe guidelines (a checklist) designed to assist researchers when reporting key aspects of meta‐ethnography (France et al., 2019), the seven phases were addressed—Phase 1: getting started, identifying the focus area and the aim of the review; Phase 2: deciding what is relevant to the initial interest; Phase 3: reading studies; Phase 4: determining how studies are related; Phase 5: translating the studies into one another; Phase 6: synthesising translations and Phase 7 expressing the synthesis—guided this synthesis. All authors contributed to the review process. The reporting of the review followed the statement to enhance transparency in reporting qualitative evidence synthesis (ENTREQ) guidelines as recommended by the Equator Network (Enhancing transparency in reporting the synthesis of qualitative research: Guidelines; Data S1; Tong et al., 2012).

### 2.2 Data collection

#### 2.2.1 Search strategy, eligibility criteria

Phase two involved conducting the literature search. A comprehensive search strategy was designed in consultation with two skilled librarians. No limitations were placed on the commencement date; the end date was 23rd August 2022. Papers were included if they reported on qualitative studies published in peer‐reviewed journals that explored immigrant and minority parent experiences of having an infant in the NICU. We only included studies of parents who differed from the majority population in the country they lived in because of their first language, culture, race or religion. Papers were also included if they focused primarily or in part on healthcare professionals' perspectives when caring for immigrant or minority families in the NICU, were written in English, and reported a primary research study.

The databases searched were CINAHL, MEDLINE, PubMed, PsycINFO, Scopus and Google Scholar, using the following terms and keywords and their derivatives: immigrant, minority families, neonatal intensive care unit, nurses and abbreviations, cross‐cultural care, refugees, ethnic minorities, and culture.

#### 2.2.2 Selection of articles

**FIGURE 1:**
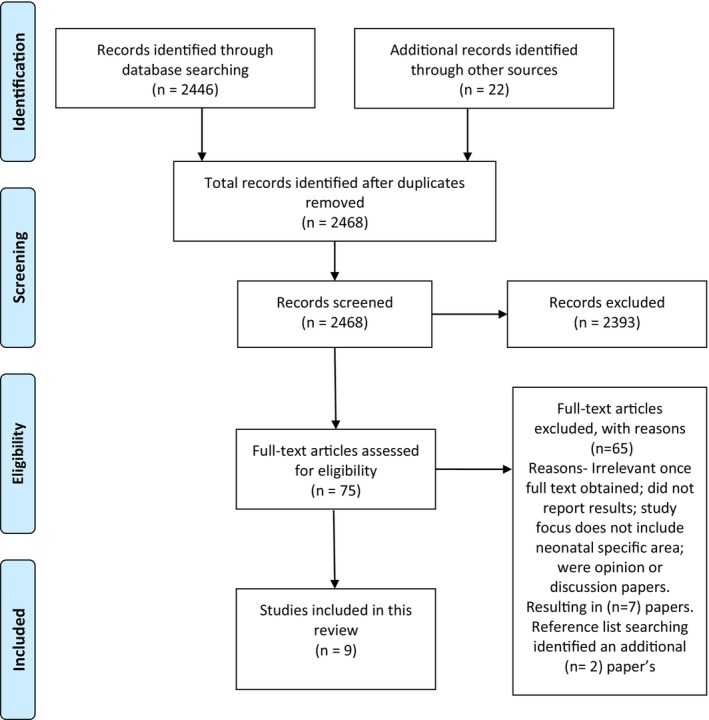
Prisma flow diagram. [Colour figure can be viewed at wileyonlinelibrary.com]

The initial search yielded 2468 articles. Duplicates were removed. Titles of all remaining papers were reviewed, and 2393 were removed (Figure 1). The abstracts of 75 papers were reviewed and excluded if they were not relevant to the topic, or they reported general experience not specific to neonatal parents or nurses and if they were quantitative studies, opinion or discussion papers (Figure 1). Twenty‐three full‐text papers for possible inclusion were then read in full by the authors. Each paper was reviewed by two authors to ensure relevance. Papers were eliminated at this stage because they did not primarily address immigrant and minority parents' perspectives or healthcare professionals' perspectives of supporting these parents in the NICU, reducing the number of articles for quality review to seven. The reference lists of these articles were searched, and an additional two articles were identified (Figure 1).

### 2.3 Data extraction, analysis and synthesis

#### 2.3.1 Critical appraisal

As all the identified articles reported findings from qualitative studies, we evaluated the rigour, credibility and relevance of the articles selected for inclusion using the Joanna Briggs Institute (JBI) Critical Appraisal Checklist tool for Qualitative research (Joanna Briggs Institute, 2020). This 10‐item checklist was used to enable the reader to identify and appraise important characteristics of the research team, study methodology, and data interpretation and analysis (Table 1). The application of the JBI critical appraisal checklist tool for qualitative research did not result in the exclusion of any articles.

**TABLE 1:** JBI critical appraisal tool.

Authors (year)	D1	D2	D3	D4	D5	D6	D7	D8	D9	D10
Ardal et al. (2011)	Y	Y	Y	Y	Y	N	Y	Y	Y	Y
Cleveland and Horner (2012a)	Y	Y	Y	Y	Y	N	N	Y	Y	Y
Cleveland and Horner (2012b)	Y	Y	Y	Y	Y	N	N	Y	Y	Y
Hendson et al. (2015)	Y	Y	Y	Y	Y	Y	Y	Y	Y	Y
Kynoe et al. (2020)	Y	Y	Y	Y	Y	U	U	Y	Y	Y
Lee and Weiss (2009)	Y	Y	Y	Y	Y	N	N	Y	Y	Y
Nicholas et al. (2014)	Y	Y	Y	Y	Y	Y	Y	Y	Y	Y
Patriksson et al. (2017)	Y	Y	Y	Y	Y	N	N	Y	Y	Y
Wiebe and Young (2011)	Y	Y	Y	Y	Y	N	N	Y	Y	Y

Abbreviations: Y, Yes; N, No; U, Unclear; N/A, Not applicable.

#### 2.3.2 Investigating the data

In phase three of the review, each article was individually reviewed by two researchers to abstract data related to the country of origin, aim, participant sample size, methodology and methods, and key findings. A data abstraction table was developed (Table 2). Each column had distinct fields and was arranged in a logical sequence to facilitate the review and analysis process.

**TABLE 2:** Included studies.

	Author, year, country, title	Aim	Participants	Methodology & Methods	Findings
1	Ardal et al., 2011, Canada Support like a walking stick: parent‐buddy matching for language and culture in the NICU.	To explore experiences of non‐English speaking mothers with preterm, very low birth weight (<1500 g) infants and examine mothers' assessment of a peer support program matching them with linguistically and culturally similar parent‐buddies.	8 immigrant mothers (Spanish, Portuguese, Chinese and Tamil) whose first language was not English.	Exploratory qualitative approach based on grounded theory; phone interviews with open‐ended questions conducted by bilingual research assistants.	Four categories identified; (1) unanticipated crisis of preterm birth; (2) situational crisis of complex NICU setting; (3) developmental crisis of loss of healthy baby at term; (4) perceptions of parent‐buddy intervention.
2	Cleveland & Horner, 2012a, USA Normative cultural values and the experiences of Mexican‐American mothers in the neonatal intensive care unit.	To explore experiences of Mexican‐American mothers who have had infants in the NICU.	15 self‐declared Mexican American mothers who speak English and had an infant in the NICU within the past 5 years. Migrant status/residency was not collected from participants due the sensitive nature of the topic within the region.	Exploratory qualitative approach; face‐to‐face interview and a brief demographics form. Directed content analysis was used.	Results reflected the importance of 5 normative Latino culture values of (1) ‘simpatia’; (2) ‘personalismo’; (3) ‘respeto’; (4) ‘familisimo’; and (5) ‘fatalismo’.
3	Cleveland & Horner, 2012b, USA Taking Care of My Baby: Mexican‐American Mothers in the Neonatal Intensive Care Unit	To develop a better understanding of the NICU experience for Mexican‐American mothers	15 self‐reported English speaking Mexican American women that had experienced having an infant in the NICU.	Grounded theory, qualitative study. Data were collected through semi‐structured interviews for theory development	Development of theoretical model titled: ‘Taking care of my baby’ describing challenges in a fluctuating context between being supportive (making meaningful connections) to inhibitive (struggling to mother). Identifying 3 key strategies mother used to help: ‘balancing responsibilities’, ‘leaving part of me with my baby’, and ‘watching over’. Process ended with 2 possibilities of desired outcome ‘bringing my baby home’ or feared outcome of ‘losing my baby’.
4	Hendson et al., 2015, Canada Culturally and linguistically diverse peoples' knowledge of accessibility and utilisation of health services: exploring the need for improvement in health service delivery.	To examine the experiences and perceptions of healthcare providers caring for new immigrant families in the NICU.	58 healthcare providers; included registered nurses, registered respiratory therapists, registered social workers, neonatologists, neonatal perinatal fellows, admin staff, registered dietitians, and students.	Exploratory qualitative, grounded theory; interviews cross disciplinary focus groups.	Overarching construct of fragile interactions associated with caring for a new immigrant family in the NICU. Factors such as crisis, decision making, differing norms and beliefs, and language contributed to the fragile interactions. During transition home, unintentional stereotyping, limited time for tangible activities, and lack of intuitive perceptions of the needs of new immigrant families also affected fragile interactions.
5	Kynoe et al. (2020) Norway When a common language is missing: Nurse–mother communication in the NICU. A qualitative study.	To explore how communication between immigrant mothers and nurses take place without having a common language, and how these mothers experience their NICU stay.	8 non‐English, non‐Scandinavian speaking mothers were interviewed individually using semi‐structured interviews. 6 mother–nurse interactions were observed, and 8 nurses' experiences explored through focus‐group interviews.	Qualitative, hermeneutic semi‐structured interview approach, observation and focus group interviews	Two overarching themes: (a) information sharing and nursing guidance despite lack of a common language, and (b) communication and caregiving despite lack of a common language.
6	Lee & Weiss, 2009, USA When East meets West: Intensive care unit experiences among first‐generation Chinese American parents.	To explore experiences of first generation Chinese American parents while their infants were receiving care in the intensive care units.	25 self‐identified first‐generation Chinese American families (25 mothers, 21 fathers, and 1 grandmother); 20 from NICU and 5 from paediatric ICU. Fir‐generation was defined as not born in the US and first in the family line to live in the US.	Qualitative, phenomenology; face‐to‐face interviews with open‐ended questions.	Findings revealed seven themes: (1) perceived incompetence; (2) self‐blame; (3) blame form others; (4) lack of support in the US; (5) filial piety; (6) communication issues; (7) cultural differences.
7	Nicholas et al., 2014, Canada Connection versus disconnection: examining culturally competent care in the neonatal intensive care unit.	To explore cross‐cultural care from the healthcare provider perspective within two tertiary level NICUs.	58 inter‐professional healthcare providers; included nurses, respiratory therapists, neonatal nurse practitioners, social workers, neonatologists, neonatal fellows, dieticians, admin staff and students/trainees.	Qualitative, grounded theory; interviews using cross disciplinary focus groups.	Two core themes identified: (1) connection and (2) disconnection heavily influencing the NICU experience and interaction. Connection was associated with congruity, synergy, and ‘fit’, untimely enhancing the relationship between the healthcare provider and family. Disconnection entailed a lack of ‘fit’ and sometimes misunderstanding and/or conflict between family and healthcare provider.
8	Patriksson et al., 2017, Sweden Communicating with parents who have difficulty understanding and speaking Swedish: An interview study with health care professionals.	To explore the experiences of healthcare professionals in Swedish neonatal care units regarding communication with parents with limited Swedish proficiency.	60 healthcare professionals; 10 physicians (7 neonatologists and 3 paediatricians), 25 nurses (2 midwives, 1 primary health nurse, 3 general nurses, and 19 paediatric nurses), and 25 nursing assistants.	Qualitative, Individual open‐ended interviews. Analysis using inductive content analysis.	One major theme identified ‘Powerlessness in the face of inadequate care routines leading to failure to communicate.’ This encompasses three sub‐themes: (1) ‘inability to perform their work properly’, (2) ‘finding their own strategies’, and (3) ‘dependence on others’.
9	Wiebe & Young, 2011, Canada Parent perspectives from a neonatal intensive care unit: a missing piece of the culturally congruent care puzzle.	To explore culturally diverse families' perceptions of culturally congruent care in the NICU of a tertiary care hospital.	21 parents; 6 Aboriginal, 3 African, 3 Vietnamese, 3 Eastern European, 2 Mixed couples (African and other), 1 South Asian (Pakistan), 1 Chinese, 1 Mennonite, and 1 Hispanic. 13 families were identified as immigrants and 8 as Canadian‐born.	Exploratory qualitative; face‐to‐face interviews approximately 1 week before discharge. 16 of the interviews were in English and 5 with a trained interpreter. 13 parent interviews, 7 with mother only, and 1 with only the father.	Four key constructions were identified and the need for: (1) provider‐client relationship of caring and trust; (2) respectful and appropriate communication; (3) culturally responsive and accessible social and spiritual supports; (4) welcoming and flexible environment.

Each article was then reviewed for content, theoretical approach and meaning of themes to inform the coding framework. Key findings and concepts were summarised for each study using raw data for the initial extraction of main findings of the study. The first reviewer used NVivo software to code each publication, the second reviewer checked the completeness of coding and the third reviewer checked for consistency and accuracy of the coding. In Phase 4, how studies related to each other was determined (first‐order interpretations) identifying key concepts and the formation of themes.

The research team first reviewed the data abstraction table (Table 2) to determine the relationship between the study aims and research questions, the study design and methods including participant groups and the key findings. During this search an index article was determined, and we developed an initial coding framework based on that article. All Authors then applied the coding framework to all articles and extracted second‐order data (authors identified themes, sub themes and interpretations) into an NVivo file for consistency and accuracy of the coding. First‐order data (quotes) illustrating each sub‐theme, theme or key finding were also extracted and coded at this time (Table 3).

**TABLE 3:** Exemplar of first and second order data.

Author	Second order data	First order – Exemplar quotes
Wiebe and Young (2011)	*Communication: Respectful and Appropriate*	‘The nurses are very accommodating and go out of their way to try to get someone who speaks French, I was very touched’ (French‐speaking African mother)
	*Provider–Client Relationship: Caring and Trust*	‘They looked after our baby like their own child. She [the nurse] made us feel safe’ (Russian mother) ‘They make us feel very, very comfortable that she [the baby] is safe’ (Vietnamese mother)
		‘The strongest theme that emerged from the interviews with parents was the importance of feeling that NICU staff and physicians genuinely cared about their infant’ (Authors – description)
Cleveland and Horner (2012b)	*Simpatia*	‘When I would come in, she [the nurse] made sure that the moms had a rocking chair. She made sure we were comfortable. She would ask if we needed anything to drink because I would stay in there for the whole 2 h at feeding time’ (Mother)
		‘Kindness and courteous interactions were important to the mothers in this study. Being made to feel welcome in the NICU was essential for the participants and they described actions taken by the nurses that helped them feel welcome’ (Authors statement)

In phase 5, the themes, concepts and key findings from each study were then translated into each other to form the new themes arising through our analysis (third‐order interpretations). Extracted themes and subthemes were reviewed again to confirm how they related or differed to one another for example, were the themes comparable known as ‘reciprocal translation’, or did they oppose each other—‘refutational translation’ (France et al., 2019). Based on the themes reported in the original studies and the emerging new themes, our synthesis was most suited to reciprocal translation. In phase 6, the research team confirmed the third‐order interpretations of the data. In phase 6, we finalised the overall textual synthesis and considered how health services and professionals can use the findings to optimise care for immigrant and minority families in the neonatal context.

## 3 RESULTS

Nine articles reporting the findings of eight studies were included in this meta‐ethnographic review. Two articles reported findings from one study (Cleveland & Horner, 2012a, 2012b). The studies were conducted in Canada (5 studies), Norway (1), Sweden (1 study) and USA (3 studies) and included a total of 279 participants (87 parents and 192 health professionals).

**FIGURE 2:**
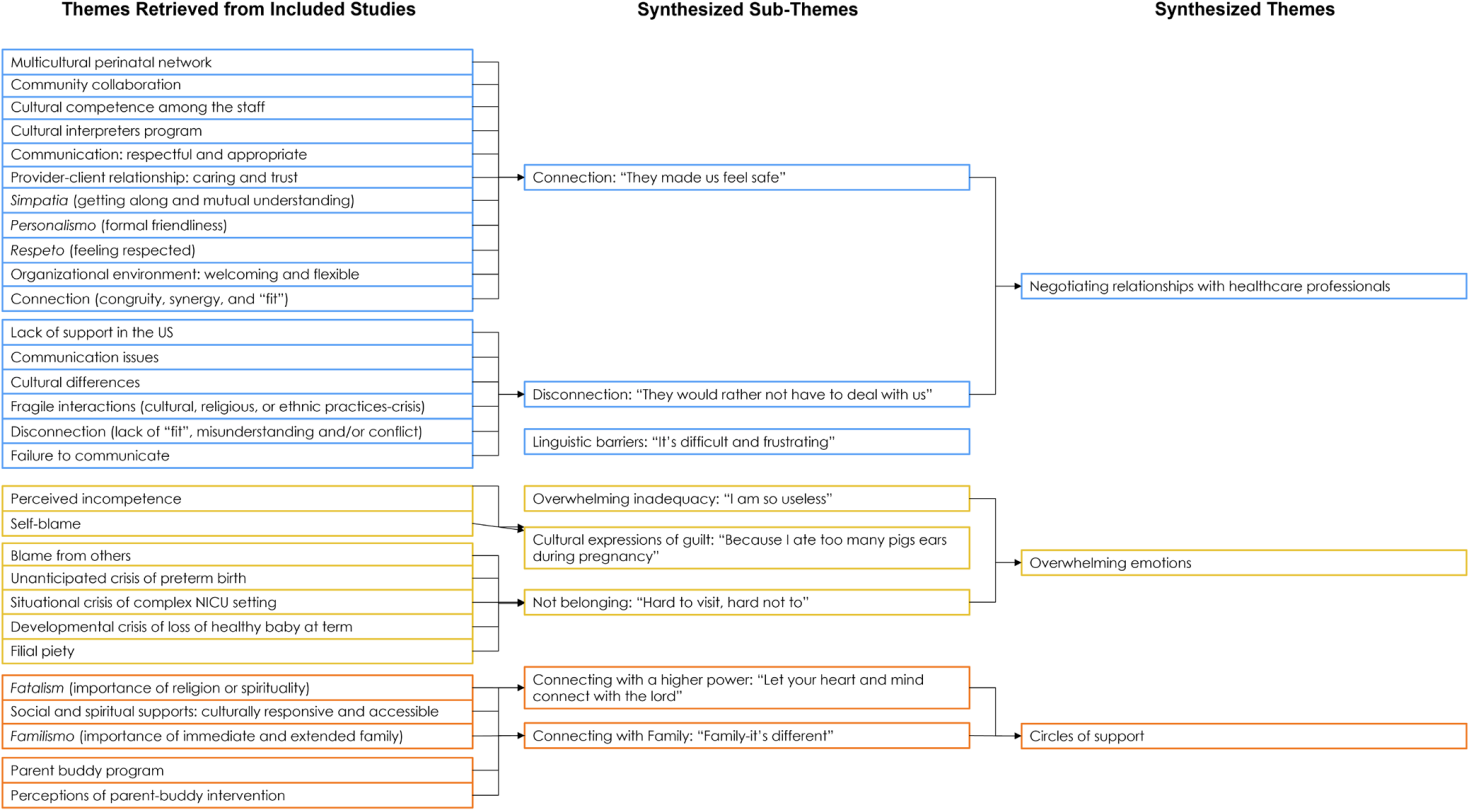
Summary of synthesis and themes. [Colour figure can be viewed at wileyonlinelibrary.com]

This meta‐ethnographic review identified three themes: (1) overwhelming emotions, (2) circles of support and (3) negotiating relationships with healthcare professionals (Figure 2). The stressful nature of having an infant admitted into the NICU for parents was evident in the included studies and is captured in the theme ‘overwhelming emotions’. Several strategies utilised by parents to cope with this unexpected experience were reported and are reflected in the theme, circles of support. Lastly, healthcare professionals play a crucial role in the NICU and can have both positive and negative influences on parents' experiences during their infant's admission in the NICU, this is captured in the third theme, negotiating relationships with healthcare professionals.

### 3.1 Overwhelming emotions

**FIGURE 3:**
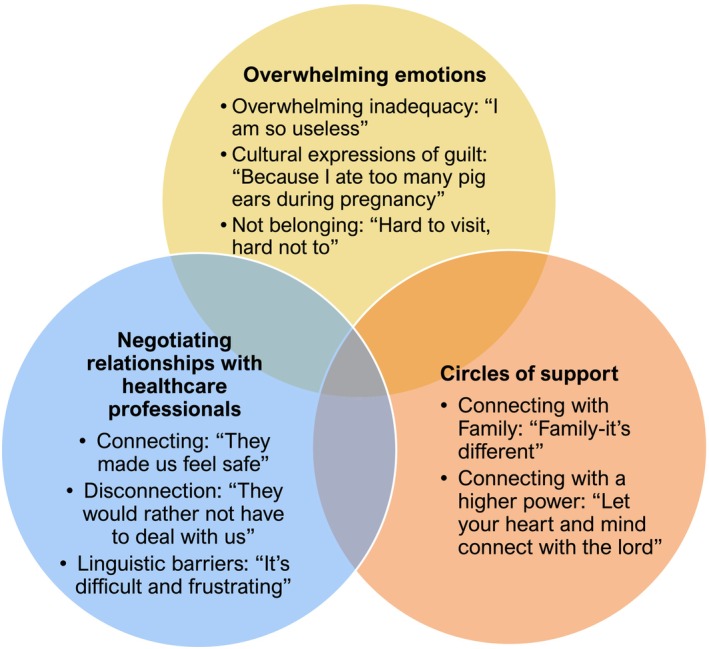
Themes diagram and attached three versions of the same diagram in different formats for you to use one of them (which whichever is easiest for you). [Colour figure can be viewed at wileyonlinelibrary.com]

One of the core themes emerging from the review of the qualitative studies on immigrant and minority families in the NICU is overwhelming emotions. This theme is comprised of two sub‐themes; *overwhelming inadequacy: ‘feeling useless as a parent*’ and *not belonging: ‘hard to visit, hard not to’* (Figure 3).

Parents in these studies reported elevated levels of uncertainty from the unexpected nature of a preterm birth or birth of a sick infant giving rise to a complex array of emotions such as anger, fear, anxiety, guilt, and helplessness. The intensive care needed for the infant provided by NICU staff shifts the natural responsibilities of parents as the primary caregivers. This leaves parents with a sense of not fulfilling their parental role. This is further exacerbated by family members blaming parents for their infants' condition and the foreign environment of the NICU.

#### 3.1.1 Overwhelming inadequacy: ‘I am so useless’

Participants in the studies had an overwhelming perception of incompetence as a parent. This stemmed from parents feeling a sense of powerlessness or inability to care for their infant in the NICU (Cleveland & Horner, 2012a, 2012b; Lee & Weiss, 2009; Wiebe & Young, 2011). This is exemplified in the following quotes ‘most of the time, I feel I am very useless because I cry in the unit…I never see anyone cry in here. I could not do anything for my son. I am always so afraid to hurt him*’* (Lee & Weiss, 2009, p. 270). This lack of confidence is further demonstrated by relinquishing control to nurses ‘The one thing l can do for her is to cooperate with the nurses and let them do whatever they want to do. I am so useless, a mother who cannot take care of her own child’ (Lee & Weiss, 2009, p. 270).

#### 3.1.2 Cultural expressions of guilt: ‘Because l ate too many pig ears during pregnancy’

Additionally, parents blamed themselves for their infants situation, and this was often associated with feelings of guilt (Ardal et al., 2011). Parent's believed that they have failed to take adequate precautionary actions, particularly failure to adhere to traditional or cultural prenatal care practices, as the underlying cause for their infant's poor health and NICU admission (Lee & Weiss, 2009). This is demonstrated in a quote from a mother of a preterm infant stating ‘At the beginning you feel guilty,’ and ‘I felt that it was my fault’ (Ardal et al., 2011, p. 94).

This self‐blame was not only reported by mothers but was also experienced by fathers. For example, in Chinese culture, as the head of the family the father is expected to maintain the health of the family (Tsai et al., 2021), as such when the child is ill, they often blame themselves for this dire outcome (Lee & Weiss, 2009).

I [father of infant in the NICU] am the person who should be responsible for my son's medical problems. I work in a laboratory where I need to handle chemical materials…I believe it was all because of me that my son has a congenital defect. (Lee & Weiss, 2009, p. 270)

Self‐blame was also a result of not meeting the expectations of others, particularly family. Lee and Weiss (2009) present an insight into filial piety, a significant Chinese virtue of respecting one's parents, elders, and ancestors. One of the core acts of filial piety includes carrying on the family's bloodline with the birth of a healthy son (Tsai et al., 2021). In the case where the infant is severely ill, some Chinese parents felt they failed to uphold their parental duty and show filial piety to their parents ‘I was very relieved to have a son for my husband and his family; but on the other hand, I also felt very ‘un‐filial’ [i.e. a bad daughter in law], because he is a premature baby’ (Lee & Weiss, 2009, p. 271).

The sense of doing the right or wrong thing by family was evident, but only reported in one of the included studies. In some instances, participants experienced verbal abuse from family members such as the in‐laws or husband, of accusing the mother as incapable of giving birth to a healthy full‐term child (i.e. in the case of a preterm birth) or failure to adhere to traditional prenatal care (Lee & Weiss, 2009). One mother described how she was blamed for her premature infant's extra ear tag stating ‘I had a fight with my sister‐in‐law the night before my labor began… My husband repeatedly told me that my daughter's ear tag developed because I ate too many pig ears during pregnancy’ (Lee & Weiss, 2009, p. 271).

#### 3.1.3 Not belonging: ‘Hard to visit, hard not to’

For most parents, the NICU environment was foreign, and it was not their space. This led to high levels of uncertainty about their parental role. In a study exploring the cultural values of Mexican‐American mothers, Cleveland and Horner (2012a) reported environmental factors such as the limited space of the NICU contributed to mothers feeling as a burden on nurses. For example, the continuous need for mothers to reposition their chairs while NICU staff were performing medical examinations was reported to make mothers feel they were in the way, ‘I'd have to move my chair here, move my chair there, or completely move my chair out of the way because it was so crowded in there. It was just so crowded, and there was no room’ (Cleveland & Horner, 2012a, p. 122) and ‘They would rather just take care of him [the infant] and not have to deal with us’ (Cleveland & Horner, 2012a, p. 170).

For participants who are experiencing acute care for the first time, this can affect their level of comfort with the NICU as well as their interactions with healthcare professionals. Immigrant parents commonly draw comparisons between the high quality of care their infant is currently receiving with that available in their country of origin (Wiebe & Young, 2011). While parents have been reported to feel gratitude for this, this was also a potential barrier for parents, preventing them from openly asking questions or expressing concern (Nicholas et al., 2014). This is exemplified by a quote taken from a healthcare professional:

I had one family who said, ‘you know, in our home, babies born before this gestation just die.’ She was terrified to even come up and see this infant because this infant was really not supposed to be alive… it took her weeks and weeks to really work that through. (Nicholas et al., 2014, p. 144).

Additionally, Ardal et al. (2011) identified the struggle mothers faced between the difficulty of visiting the NICU while also their desire to be at home: ‘It's hard to be visiting and hard not be visiting’ (p. 93).

### 3.2 Circles of support

The second core theme identified the importance of circles of support including support from their family, connecting with their spirituality or religious beliefs, and social support from their friends or peers.

#### 3.2.1 Connecting with family: ‘Family–it's different’

Family was reported as a crucial source of both emotional and care support for the mother and infant for both during their admission to the NICU and post‐discharge support. Wiebe and Young (2011) further support the need of family for culturally diverse parents, as they identified family as the most vital form of social support: *‘*My sisters, his aunties and my grandmother all come to help me’ (p. 81). The traditional roles that family plays in the event of birth (e.g. the educational support offered by grandparents on cultural practices for taking care of the infant), also contributes to the need for family as a form of emotional support.

Cleveland and Horner (2012a) noted that the most significant difficulty reported by Mexican‐American mothers was leaving the infant alone at the NICU. Mothers highlighted that leaving various family items such as family pictures, infant clothing, and a blanket with their infant helped them cope with the stressful experience of separation from their infant while in the NICU. These gestures helped parents to feel that they were able to provide comfort and ‘warmth’ for their infant in contrast to the ‘cold’ NICU equipment and material:

It's a mindset. We feel more at ease being there, seeing things from home, it makes us feel more at home with the infant, more in a homey place. I was glad to see my little girl's picture [on her son's bed]. (Cleveland & Horner, 2012a, p. 123)

Mothers also reported the desire to connect with nurses in a personal but professional manner. This, at times, could substitute for family not being there. As during the course of the infant's hospitalisation in the NICU, nursing staff would eventually meet the mother's immediate family, while the mother may not know anything other than the nurse's name. Given the vulnerability parents feel while in the NICU, discussing everyday activities with staff outside the scope of the NICU contributed to a positive experience in the NICU:

It meant a lot because I'd be there all day, so we would talk about their personal things. It felt good because she knew all of my children, all of my family, so now I know hers, and we were bonding. (Cleveland & Horner, 2012a, p. 122)

When we walk in, they [the nurses] say, ‘Come here, Dad, how are you, and how is Mom? Come to see the baby, the baby's doing well.’ We don't have to ask, they just tell us, and ask if we have any questions. They made us feel very, very comfortable that she [the infant] is safe. (Wiebe & Young, 2011, p. 79)

Overall, mothers valued the nursing staff's capacity to connect with them over their abilities to perform medical procedures, as it wasn't within their capacity to measure the professional competency of staff but rather their social interactions with them (Wiebe & Young, 2011).

Furthermore, in the case of post‐discharge support for the infant, mothers noted that having family members nearby was crucial. Factors such as living long distance from the hospital and first‐time mother status, further increase the need for family support (Cleveland & Horner, 2012a). Mexican‐American mothers also reported the value of family over friends for post‐discharge support, noting that they felt both relying on family and the level of care the infant will receive would be a far superior choice than friends (Cleveland & Horner, 2012a). The negative impact on immigrant parents of separation from their family is exemplified in the following quotes: ‘My husband and I are the only ones who live here in Canada … It was five months of, let's say, agony, torture and very sad,’ and ‘We don't have anybody here, not even my parents’ (Ardal et al., 2011, p. 94).

The lack of support impacts significantly on families and many are not aware of any form of social support resources available to them (Lee & Weiss, 2009; Nicholas et al., 2014).

In the study by Ardal et al. (2011), culturally diverse parents were interviewed about their experiences of a peer‐to‐peer support intervention for mothers with infants in NICU. They reported that this peer support program validated their feelings and helped to alleviate feelings of guilt and anxiety. They were seen as providing a trustworthy source of information and facilitating a sense of empowerment. The need to culturally match the support ‘buddy’ with the mother and the need for a common language were vital to the efficacy of the peer‐to‐peer support program (Ardal et al., 2011; Nicholas et al., 2014). One participant in the study by Ardal et al. (2011) stated ‘Yes, we have a common language; we bring up kids with the same customs; to be able to communicate using the same language is very important’ (p.94).

#### 3.2.2 Connecting with a higher power: ‘Let your heart and mind connect with the Lord’

On an individual level, spirituality among culturally diverse and immigrant parents plays a key role in their ability to cope with a stressful situation and spiritual practice as means of asking for good health (e.g., welcoming infant ritual). Wiebe and Young (2011) reported Aboriginal and immigrant parents noted the importance of prayer, visiting a place of worship, and seeking direction from spiritual leaders in aiding their ability to deal with their infant in the NICU. Connecting on a spiritual level to a higher power also aided the process of acceptance with serious illness and with death. Several Mexican‐American mothers highlighted their belief that their child's condition was fated, and while the need for time to practice spiritual beliefs was emphasised, this was reported to eventually facilitate their acceptance of the dire situation (Cleveland & Horner, 2012a):

You have to let your heart, and your mind connect with the Lord. I have a close bond with Jesus Christ. We have to give ourselves time to get any miracle that he would have for us. After all my prayers, he started getting worse again, so I knew, it's time to let go, this is just giving me preparation. (Cleveland & Horner, 2012a, p. 123)

### 3.3 Negotiating relationships with healthcare professionals

Given the primary role of healthcare professionals in the NICU as caregivers for the infant, their various interactions with parents can have a profound effect on their experiences and attitude towards the NICU. As such, negotiating relationships with healthcare professionals was a common theme in each of the studies (Figure 2. Summary and Synthesis of Themes). This theme is centred around building a respectful and professional‐friendly relationship with immigrant or minority parents, establishing a sense of trust and care for the infant's wellbeing, and maintaining clear and transparent communication. All of which contribute to positive connection between healthcare professionals and immigrant and minority parents. Participants also identified personal barriers such as language, events or interactions that resulted in feeling disconnected.

#### 3.3.1 Connecting: ‘They made us feel safe’

It is through warmth, rapport, and empathy that healthcare professionals can facilitate a respectful and professional relationship with parents. This forms the core values of the sub‐theme, connecting: ‘they made us feel safe’. Mexican‐American women used this phrase in the study by Cleveland and Horner (2012a) to exemplify the importance of staff who made them feel welcomed in the NICU, demonstrated through smiling, greeting, and recognising the arrival of mothers (Cleveland & Horner, 2012a; Wiebe & Young, 2011). Such simple gestures were reported to express a sense of warmth and caring. A non‐judgmental environment is also associated with a welcoming one, particularly reported by vulnerable groups of mothers (e.g., ex‐drug users) failure to do so may result in mothers feeling unwelcome and adding stress when visiting the NICU (Cleveland & Horner, 2012a).

Parents who felt their healthcare professional genuinely cared for the health and wellbeing of their child facilitated trust, confidence, and a sense of security (Wiebe & Young, 2011). Parents generally evaluated the level of care provided by healthcare professionals by paying careful attention to how NICU staff interacted with their infant through verbal and physical demonstration. For example, reacting promptly when the infant cries, ensuring the infants hygiene and nourishment needs are met, taking the time to personally connect with parents and teaching parents various care instructions for the infant (Wiebe & Young, 2011). This is yet another indication of how immigrant parents measure the quality of care the infant is receiving through social and personal interactions versus the technical (or medical) expertise of NICU staff (Wiebe & Young, 2011). Wiebe and Young (2011) showed that parents emphasised the need to feel that health professionals genuinely cared for the well‐being of their child. One parent stated, ‘They looked after our infant like their own child. She [the nurse] made us feel safe’ (Wiebe & Young, 2011, p. 79).

It is noteworthy that rotating staff shifts were repeatedly reported to hinder the capacity of parents to trust staff (Hendson et al., 2015; Wiebe & Young, 2011). In the case where parents did not have a trusting relationship with their healthcare professional, they tended to pay closer attention to the care delivered by staff and minor incidences, or misunderstandings were often not pardoned by parents. This may lead to conflict between parents and healthcare professionals (Nicholas et al., 2014; Wiebe & Young, 2011). From the healthcare professional perspective, taking the time to understand the cultural norms in a genuine manner and establishing a personal connection with the family helped to build a positive relationship between parents and healthcare professionals (Hendson et al., 2015; Nicholas et al., 2014).

#### 3.3.2 Disconnected: ‘They would rather not have to deal with us’

An understanding of the need to practice cultural customs was also noted by mothers and having health professionals understand this was appreciated. Conversely, when this was not the case, it often resulted in disconnection between healthcare professionals and parents, as reflected by this subtheme disconnected: ‘they would rather not have to deal with us’. For example, in Chinese culture, mothers are expected to adhere to a diet consisting of food believed to benefit the mother and aid in the production of breastmilk. Cultural practices may also apply to the post‐partum period, such as the Zuo‐Yuezi (‘sitting the month’). From the healthcare professional perspective, one participant stated:

In some cultures, it is common for the mom to stay inside at home for one month after delivering a newborn baby… we often question why a mom is not visiting and isn't participating in the care. It is really easy to forget all these difference cultural norms. (Nicholas et al., 2014, pp. 145–146)

From the healthcare professional perspective, several cultural norms that are implicitly expected of NICU staff by family and community members have also been reported to cause issues with parents. Nicholas et al. (2014) provide insight into how several aspects of cultural norms and factors such as the gender of the healthcare professional, mothers' clothing choices, cultural perspectives on medicine, treatment planning, and leaving decision‐making to health professionals or extended family, may impact on the interactions between healthcare professionals and parents (Hendson et al., 2015; Nicholas et al., 2014). For example, some immigrant mothers expect that only same‐sex healthcare professionals should communicate with them. Failure to adhere to such cultural norms may result in offending the parents or cause a misunderstanding (Nicholas et al., 2014).

Given the complexity of facilitating culturally competent care within the NICU and the many barriers associated with it, communication issues are a common challenge described in the literature. This includes non‐verbal communication concerning body language, tone, and volume. For example, staff raising their voices when speaking and physical gestures such as finger‐pointing can be perceived negatively by parents and may contribute to creating a stressful and uncomfortable environment. ‘It creates more stress when we're seeing our son in very bad condition and then the nurses not being nice, like their tone of voice, what they would say, and how they present themselves’ (Wiebe & Young, 2011, p. 80). The various policies enforced within the NICU were a common complaint by mothers, such as not allowing parents to sleep beside their infant, limited visiting hours, and lack of privacy while breastfeeding (Wiebe & Young, 2011).

#### 3.3.3 Linguistic barriers—‘It's difficult and frustrating’

Language and communication were also a major concern for both parents and healthcare professionals, often resulting in parents not understanding the condition of their child, causing parents to feel anxious, and both healthcare professionals and parents feeling frustrated due to the miscommunication and difficulty in understanding each other (Nicholas et al., 2014; Patriksson et al., 2017; Wiebe & Young, 2011). This is further illustrated in the following quote from a healthcare professional. ‘We are trying to help, and sometimes you can't cope; you've shown them everything and repeated yourself, and it's difficult and frustrating for the mother, and frustrating for the staff, so sometimes you almost give up’ (Patriksson et al., 2017, p. 3).

Parents valued staff taking the time to communicate in the language they are most comfortable with. This indicated that the staff cared (Wiebe & Young, 2011). In situations where communication was not clear, often due to language barriers, parents were hesitant to ask questions. Additionally, some parents believed the healthcare professional was withholding information from them (Patriksson et al., 2017; Wiebe & Young, 2011). Healthcare professionals also felt they were not able to effectively perform their duties or adhere to the standard procedures of their profession and felt a general sense of discomfort:

Sometimes you feel like you are just not reaching the full potential of care if you are trying to communicate with the parents [regarding] how to do something for the baby and they are not quite understanding. You are kind of limiting how far they can go in the hospital setting. (Nicholas et al., 2014, p. 145; Patriksson et al., 2017)

Overall, both parents and healthcare professionals felt a lower quality care and less‐optimal experience with the NICU was a barrier to enabling a positive relationship between NICU staff and parents. Patriksson et al. (2017) described a number of approaches staff took upon themselves to help both parents and staff feel understood. ‘You have to kind of show them and point, and your gestures get more sweeping, your whole body and perhaps even facial expressions, you, yes, you reinforce some things’ (Patriksson et al., 2017, p. 3).

Kynoe et al. (2020) reported communication aids such as glossary term tables did not contain adequate information for nurses when communicating with parents on a daily basis, and technological devices such as translating apps were viewed as unreliable. While interpreters were used with consultations with physicians, they were rarely used for nurse‐parent interactions (Kynoe et al., 2020):

We do not think as often as we should that, ok, if mother does not speak Norwegian, then we will book an interpreter (Kynoe et al., 2020, p. 2226).

While language barriers made it difficult to assess mothers understanding, mothers did report feeling adequately informed and included in their infant's care.

The use of interpreters with culturally diverse and immigrant parents appears to be associated with both positive outcomes, such as parents understanding the information given by healthcare professionals, but also negative outcomes stemming from the over‐reliance placed on interpreters to overcome the language barrier. This includes limitations in professional translator availability, complications in parent's interpretation of the information that may result in miscommunication, and the limited use of interpreters in the NICU (Patriksson et al., 2017; Wiebe & Young, 2011). Several self‐identified limitations were also highlighted by healthcare professionals, such as unintentional stereotyping, difficulty in incorporating the extra time needed to carry out intangible activities (e.g., establishing a personal relationship with parents), and lack of instinctive ability to recognise the family's unique needs (Hendson et al., 2015).

## 4 DISCUSSION

The findings of this meta‐ethnographic study of immigrant and minority parents' experiences in the NICU demonstrated that such families face unique stressors. The experience of having an infant admitted to the NICU by any parent is characterised by overwhelming emotions of fear, shock, and guilt at the same time as holding strong feelings of love for the infant. Immigrant and minority parents' distress can be buffered by access to or ability to perform cultural rituals associated with having a baby and having access to a support network or a circle of support. The stress and distress felt by parents is exacerbated when there are language barriers and or the health services are not able to meet parent's specific needs, and the parents must carefully negotiate their place in the NICU and relationships with staff. Healthcare professionals were also challenged to provide family‐centred care because of limited resources, staff access to interpreters and cultural mediators, and a lack of multicultural policy advice.

The first theme, overwhelming emotions reflects the high levels of distress, depression, and anxiety that immigrant and minority parents with an infant in NICU report. These experiences are well‐known in relation to parents of infants in NICU, who often struggle to find a sense of identity as a parent and to feel they belong in the NICU (Thomson et al., 2020). Feeling cared for, respected and valued is considered important for creating a sense of belonging and social connectedness (Thomson et al., 2022). Parents liked when staff asked them how they were doing as this demonstrated an overall sense of caring (Cleveland & Horner, 2012b). Having a sense of ‘not belonging’ in the neonatal intensive care unit also mirrors the feelings that immigrant parents have described as they transition to parenthood in a new country. For example, in a meta‐ethnographic review (Pangas et al., 2018) described how refugee women felt as though they were living between two cultures and that pregnancy birth and new parenthood often caused many to evaluate their cultural beliefs and their identity as a citizen and mother in the new country.

In this review, some parents found ways to overcome the feeling of alienation or separation from their infant for example, Cleveland and Horner (2012a) report Mexican‐American mothers adapted to the situation by displaying various objects representative of the family and home. Mothers viewed this practice as highly symbolic and meaningful, as it created a sense of comfort and connection with the family. In a recent integrative review of the cultural determinants that influenced parents' experiences of neonatal care Nyaloko et al. (2023) and colleagues highlight the importance of artefacts described as ‘symbols of fortune’, that families believe bring good fortune and protection. Objects such as bracelets, necklaces, or symbols are used to ward against evil spirits (Nyaloko et al., 2023). Other findings reported were that cultural beliefs and practices influence parent infant‐rearing practices, and health professionals need to understand various cultural determinants to provide culturally sensitive care specific to the needs of infants and families in their care (Nyaloko et al., 2023).

Parents also valued receiving information about their infant and were pleased when staff shared information or offered education so that they could more competently care for and advocate for their infant. Parents also report accurate written material in their language can limit the need for an interpreter (Batton et al., 2023). Thomson et al. (2020) report parents should receive regular information (verbally, written, or digitally) about their infant and support services available, and describe that when parents feel informed about the care of their infant, they build a closer sense of connection.

In our review, the theme ‘overwhelming emotions’ also highlights how distinct sources of stress particularly related to cultural values and beliefs can impose an additional emotional burden on immigrant and minority parents. Similarly, Nyaloko et al. (2023) emphasised that neonatal staff should recognise that culturally diverse parents may want to engage in spiritual care practices including intragenerational infant‐rearing practices, and opportunities to combine traditional practices with treatment.

In some countries, senior family members maintain a position of power and influence over the family and child‐rearing practices. For example, attending to daily activities, bathing infants, and attending to care even if it conflicts with current medical practices (Nyaloko et al., 2023). Lee and Weiss (2009) reported that Chinese parents experienced the burden of the culturally imposed expectations to respect their parents, elders, and ancestors through the birth of a healthy child to carry on the family bloodline. As a result, Chinese parents may believe they have failed to uphold their duties of carrying on the family bloodline because of the poor health condition of their child and the possibility of developmental delay in the child. Ardal et al. (2011) reported that mothers were quick to accept blame for the infant's premature birth. Recent research has elaborated on the shame and suffering experienced by the Taiwanese grandmothers of children with a disability (Huang et al., 2020). Huang et al. explain that in cultures influenced by Confucianism, there is the individual‐self, and the greater‐self, which includes family members and significant others; and both are part of one's self‐identity. Therefore, it is not only the parent that will experience shame, but these parents are also aware that they will be transferring shame to their own parents (Huang et al., 2020).

The second theme circles of support demonstrates that immigrant and minority parents, like all families, rely strongly on their family and relatives to help them cope with this stressful situation (Cleveland & Horner, 2012a; Wiebe & Young, 2011). Immigrant and some minority parents, however, may not have access to such crucial sources of support with families and friends living overseas and they can experience greater distress having to cope alone and are therefore more likely to experience perinatal mental health problems such as postnatal depression (Dela Cruz et al., 2023). For example, studies in this review report women feeling lonely, isolated, and devalued often relying on their partner, interpreters, or health professionals for emotional support and information (Lee et al., 2009). Furthermore, a common misconception is that minority groups have larger families and can offer informal support; however, acculturation, a process where minority cultures gradually adopt the ethos and values of the majority culture has been reported to contribute to the erosion of traditional support networks (Redman et al., 2024).

Fathers of premature infants also had to take on a greater role in helping to support the mother in absence of her close family. This places a greater burden or higher expectation on male partners. Men, who have typically grown up with certain perceptions of the father role, now face the situation where they have to act as a substitute for the extended female family members. Many studies of immigrant women report how much they rely on their partner as the main support person in the perinatal period (Lee et al., 2009). For example, Taiwanese mothers undertaking a month lying‐in period relied on their partner or family members as a valuable source of information, help and support (Lee et al., 2009). This can place significant pressure on men to take on a new role, particularly in the context of NICU as many cultures do not have fathers actively participate in the care of infants (Nyaloko et al., 2023). Research indicates that immigrants experience role reversals in families between gender, parents, and children (Wali & Renzaho, 2018). An Australian study reports immigrant men are having to shift from a more nuclear family model to men taking on a larger supportive role in comparison to their role in their home country (Forbes et al., 2021). Additionally, there are accounts of parents attempting to recreate an extended family network through close friends they described as family (Forbes et al., 2021). However, it can or may be difficult to draw on these new networks when the infant is in NICU for prolonged periods of time, and the mother and father are less able to connect with their new community.

Interactions between healthcare professionals and immigrant and minority parents were the strongest recurring theme from the included studies of this meta‐ethnographic investigation. Both positive and negative interactions were reported from parents and healthcare professionals. Almost all emphasised the importance of developing respectful partnerships, negotiating roles, and acknowledging that each family is unique is important when caring for infants and families in the NICU. The importance of parent‐nurse or health‐professional relationships in the NICU has been emphasised in many studies. Health professionals act for all intents and purposes as the gatekeepers in the NICU facilitating access to or inhibiting parent access to infants (Flacking et al., 2022; Naylor et al., 2020). In the meta‐ethnographic review by Thomson et al. (2020), neonatal staff were crucial in facilitating the closeness between a parent and their infant. Spending uninterrupted quality time with the infant was considered important for emotional connections between the infant and parent (Thomson et al., 2020).

In this review, health professional accounts about caring for immigrant and minority families in the NICU revealed complex relationships and fragile interactions (Hendson et al., 2015; Nicholas et al., 2014; Patriksson et al., 2017). For example, Hendson et al. (2015) highlight unintentional stereotyping, lack of intuitive perceptions of the needs of new immigrant families and time limitations for intangible activities. There appears at times to be a clash between the values of healthcare professionals and immigrant and minority parents. The dynamic, multifaceted concept of culture is not limited to ethnic identity or shared heritage but also the shared meanings and understanding of a group's beliefs and practices, which are fluid and can change at any time.

This highlights the need for further education of staff and research exploring the experiences of culturally diverse parents within the NICU to foster understanding and achieve the goal of family‐centred care (Trajkovski et al., 2012, 2016).

### 4.1 Strengths and limitations

The strengths of this meta‐ethnography review are the systematic approach used and synthesis of information on immigrant and minority parents' experiences of having a newborn infant in the NICU and healthcare professionals caring for these families. However, limitations of this review are that the pool of studies was low and only full‐text articles available in English were included in this review, and other studies not written in English have not been included. Most of the included articles reported their findings from hospitals in Canada and the United States, indicating that there is still a wide range of cultural and heath care context that need to be investigated. Additionally, articles may have been excluded if they were still in progress at the time of publication.

Given the small number of specific studies on this topic, further qualitative research is needed to explore the unique sources of stress that can impact immigrant and minority parents as well as to equip healthcare professionals with awareness and the knowledge to provide culturally safe holistic family‐centred care, welcoming parents, understanding the unsettling nature of having an infant in the NICU and implementing support services is required.

## 5 CONCLUSION

This review illustrates the wide‐ranging impact of having an infant admitted to NICU on both immigrant and minority parents and healthcare professionals. The importance of circles of support on an individual level (spirituality) and external level (connecting with family) was highlighted. Negotiating relationships with healthcare professionals and challenges of connecting, feeling disconnected, and linguistic barriers were reported. While there are mismatches between immigrant and minority families' needs and the support provided, there are also cultural and family strengths displayed by immigrant families and culturally respectful initiatives employed by NICU professionals.

## 6 RELEVANCE TO CLINICAL PRACTICE

Parents in the included studies reported many positive aspects of the care for their infants in the NICU. However, disparities continue to exist in delivering care to immigrant and refugee families, and immigrant mothers report perceived less discharge readiness when leaving the NICU (McGowan et al., 2019). Such barriers include the disconnection from traditional family support, cultural barriers creating a sense of lack of understanding, and, in some cases, communication issues due to a language barrier.

All parents require support when having an infant in the NICU. However, migrant and minority parents have unique needs and require care to be tailored to assist this vulnerable population. The health professionals who participated in these studies were aware of this higher level of need but at times were at a loss as to how best to respond (Henderson & Kendall, 2011; Kynoe et al., 2020). Given the limited access to family and friends, it is worth exploring ways to implement a peer‐to‐peer support system such as connecting people from similar backgrounds or cultural beliefs. There is increasing evidence of the benefits of supporting families including peer‐to‐peer support in a variety of situations results in more empowered, confident, and adaptive parents and assists in reducing parental stress, anxiety, and depression (Ardal et al., 2011; Hall et al., 2015).

It is also important to ensure that healthcare staff are well educated and have the skills to communicate with immigrant and minority parents in a culturally sensitive way. Social workers have an important role, and this service may not be familiar to immigrant and minority parents. Additional support from social workers may be required for families with financial constraints, visa access, employment, housing, and access to health care in the new country.

## AUTHOR CONTRIBUTIONS

S.T., M.A., S.R., N.G., M.L. and V.S. designed the search strategy. S.T., M.A. and V.S. reviewed papers for quality and inclusion. S.T., M.A. and V.S. extracted data from papers. ST, M.A. and V.S. produced the preliminary analysis. S.T., M.A., S.R., N.G., M.L. and V.S. developed draft of full manuscript. S.T., M.A., S.R., N.G., M.L. and V.S. reviewed drafts and full manuscript for final submission. All authors contributed the study design, selection and analysis of all included articles, and agreement on final manuscript, including all tables and figures.

## FUNDING INFORMATION

We would like to acknowledge and thank Western Sydney University for the provision of Early Career Research funding.

## CONFLICT OF INTEREST STATEMENT

The authors declare no conflicts of interest.

## Supporting information


Data S1.

## Data Availability

The data that support the findings of this study are available from the corresponding author upon reasonable request.
